# Methotrexate versus cyclophosphamide for remission maintenance in ANCA-associated vasculitis: A randomised trial

**DOI:** 10.1371/journal.pone.0185880

**Published:** 2017-10-10

**Authors:** Federica Maritati, Federico Alberici, Elena Oliva, Maria L. Urban, Alessandra Palmisano, Francesca Santarsia, Simeone Andrulli, Laura Pavone, Alberto Pesci, Chiara Grasselli, Rosaria Santi, Bruno Tumiati, Lucio Manenti, Carlo Buzio, Augusto Vaglio

**Affiliations:** 1 Nephrology Unit, University Hospital of Parma, Italy; 2 Nephrology and Immunology Unit, ASST Santi Paolo e Carlo, San Carlo Borromeo Hospital, Milano, Italy; 3 Nephrology and Dialysis Unit, A. Manzoni Hospital, Lecco, Italy; 4 Nephrology Unit, IRCCS Fondazione Cà Granda Maggiore Policlinico Hospital, Milano, Italy; 5 Pneumology, Bicocca University, Milano, Italy; 6 Internal Medicine, Arcispedale Santa Maria Nuova, Reggio Emilia, Italy; VU University Medical Center, NETHERLANDS

## Abstract

**Objectives:**

The treatment of anti-neutrophil cytoplasmic antibody (ANCA)-associated vasculitis (AAV) is based on remission-induction and remission-maintenance. Methotrexate is a widely used immunosuppressant but only a few studies explored its role for maintenance in AAV. This trial investigated the efficacy and safety of methotrexate as maintenance therapy for AAV.

**Methods:**

In this single-centre, open-label, randomised trial we compared methotrexate and cyclophosphamide for maintenance in AAV. We enrolled patients with granulomatosis with polyangiitis (GPA), microscopic polyangiitis (MPA) and eosinophilic granulomatosis with polyangiitis (EGPA), the latter with poor-prognosis factors and/or peripheral neuropathy. Remission was induced with cyclophosphamide. At remission, the patients were randomised to receive methotrexate or to continue with cyclophosphamide for 12 months; after treatment, they were followed for another 12 months. The primary end-point was relapse; secondary end-points included renal outcomes and treatment-related toxicity.

**Results:**

Of the 94 enrolled patients, 23 were excluded during remission-induction or did not achieve remission; the remaining 71 were randomised to cyclophosphamide (n = 33) or methotrexate (n = 38). Relapse frequencies at months 12 and 24 after randomisation were not different between the two groups (p = 1.00 and 1.00). Relapse-free survival was also comparable (log-rank test p = 0.99). No differences in relapses were detected between the two treatments when GPA+MPA and EGPA were analysed separately. There were no differences in eGFR at months 12 and 24; proteinuria declined significantly (from diagnosis to month 24) only in the cyclophosphamide group (p = 0.0007). No significant differences in adverse event frequencies were observed.

**Conclusions:**

MTX may be effective and safe for remission-maintenance in AAV.

**Trial registration:**

clinicaltrials.gov NCT00751517

## Introduction

The anti-neutrophil cytoplasmic antibody (ANCA)-associated vasculitides (AAVs) include granulomatosis with polyangiitis (GPA, formerly Wegener’s granulomatosis), microscopic polyangitiis (MPA) and eosinophilic granulomatosis with polyangitiis (EGPA, formerly Churg-Strauss syndrome) [1]. Their histopathological hallmark is small-vessel necrotising vasculitis along with granulomatous lesions in GPA and eosinophil-rich inflammation in EGPA. ANCAs are positive in ~70–90% of patients with GPA and MPA [2], and in ~40% of those with EGPA [3].

GPA and MPA have historically been grouped together in treatment trials because of their clinical and histological characteristics and their similar response to immunosuppressive therapies, whereas EGPA has been kept separated; data on therapeutic interventions in EGPA are limited to very few trials [4–6]. During the past two decades, several studies have demonstrated in GPA and MPA the feasibility of a staged treatment approach based on remission-induction followed by maintenance therapy; this has not yet been proven for EGPA. Induction therapy is usually based, particularly in patients with generalised disease, on the combination of glucocorticoids with cyclophosphamide (CYC) or rituximab [7–9]. Several agents have been tested for remission maintenance. Azathioprine was first identified as a good option because it proved of comparable efficacy to CYC [10]. Subsequent maintenance trials demonstrated that methotrexate (MTX) and azathioprine had comparable efficacy [11] while mycophenolate mofetil was less effective than azathioprine [12]. A recent trial showed that rituximab was more effective than azathioprine in maintaining remission [13], although its costs are still high and its long-term safety remains to be ascertained. Thus, although several agents are available for remission maintenance in AAV, further data coming from controlled trials are needed. Additionally, the optimal maintenance strategy for EGPA remains to be investigated.

When we designed this trial, in 1997, no randomised studies had yet been published in AAV. The major objective of this study was to compare the efficacy and safety of CYC with that of MTX, which was widely used for autoimmune diseases. We also included EGPA patients with poor prognosis factors, as they were known to require the combination of immunosuppressants and glucocorticoids for the prevention of flares. This was a single-centre trial, and therefore suffered from a slow enrolment rate; we decided to close it (in 2011) although enrolment had not yet been completed also because of the parallel availability of novel agents for remission maintenance. Our findings support the role of MTX as a maintenance agent, provide additional data on its long-term safety, and also show, in a subgroup analysis, the feasibility of an induction-maintenance approach in EGPA.

## Methods

### Study design

This open-label randomised trial was conducted at the Nephrology centre of Parma University Hospital, Italy; the patients were enrolled between December 1997 and June 2011. The study was performed in accordance with the Declaration of Helsinki; the protocol was approved by the Ethical Committee of Parma University in February 1997 and registered in clinicaltrials.gov when trial registration became mandatory (NCT00751517). The authors confirm that all ongoing and related trials for this drug/intervention are registered.

Inclusion criteria were clinically active AAV and an age of 18–80 years. All patients with GPA or EGPA were required to fulfill the 1990 American College of Rheumatology criteria and/or the 1994 Chapel Hill Consensus Conference definitions [1, 14, 15]. Patients with MPA had to meet the 1994 Chapel Hill definitions [1]. EGPA prognosis was assessed according to the five-factor score (FFS) [16]; only patients with FFS≥1 or with peripheral neuropathy were included [5, 17]. Exclusion criteria were other systemic autoimmune/connective tissue diseases, active HBV or HCV infections, HIV seropositivity, known hypersensitivity to study medications, pregnancy, cancer, and an estimated glomerular filtration rate (eGFR, calculated using the Cockroft-Gault formula) <10 ml/min/1.73m^2^. All patients provided written informed consent.

### Treatment and assessments

All enrolled patients received three IV methylprednisolone pulses (500 mg/pulse) followed by oral prednisone and CYC. Prednisone was given at an oral dose of 1 mg/kg/day for the first month, 0.5 mg/kg/day for month 2, 0.25 mg/kg/day for month 3, and then gradually tapered to 5 mg/day by month 6. CYC was administered at an initial oral dosage of 2 mg/kg/day, which was kept stable until remission if no toxicity occurred. CYC dose was reduced for age (≥60 years) and renal function (serum creatinine≥3.4 mg/dL)[8]. Remission was defined as a Birmingham Vasculitis Activity Score (BVAS) of 0 [18] and had to be reached by month 9, otherwise the patients were excluded from the study. The patients who achieved remission were randomly assigned to receive CYC or MTX. Randomisation was performed by SA using a computer algorithm concealed from the other investigators; the patients were randomised to CYC or MTX at a 1:1 ratio. For maintenance, CYC was given at a dose of 1.5 mg/kg/day while MTX was given at an initial dose of 15 mg/week, which was increased until the dose of 0.3 mg/kg/week was reached (maximum 20 mg/week). The patients underwent randomisation only if their eGFR was>30 ml/min/1.73m^2^ and their aminotransferase levels were below 2x upper limit of normal. Patients with eGFR of 30–50 ml/min/1.73 m^2^ received 75% of the full CYC dose and half of the full MTX dose.

The patients randomised to CYC received trimethoprim-sulfamethoxazole prophylaxis (80/400 mg/day), while those treated with MTX received oral folic acid (5 mg/week). Both groups received gastric protection (proton-pump inhibitors or H2-blockers), calcium, vitamin D and/or bisphosphonates. Maintenance treatment was continued for 12 months, at the end of which MTX or CYC were discontinued; prednisone was instead continued at 5 mg/day. The patients were followed for an additional 12 months (24 months from remission). Disease response was assessed every month before randomisation and then every 3 months. Each assessment included a BVAS evaluation and routine laboratory tests [full blood counts, renal and liver function tests, C-reactive protein (CRP), erythrocyte sedimentation rate (ESR), ANCA, urinalysis and 24 hour-proteinuria]. All patients also underwent urinary cytological examination every six months during the treatment period and every year during the post-treatment follow-up, and chest radiograph and abdominal ultrasound every year during the whole study period. Adverse events were graded according to the National Cancer Institute Common Terminology Criteria for Adverse Events, version 3 [19].

### Study end-points

The primary end-point included the relapse frequency by month 12 (from the beginning of maintenance treatment) and the time to relapse. Relapse rates at month 18 and 24 were also evaluated. Secondary end-points included rates of major and minor relapses per group, change in eGFR and proteinuria, treatment–related toxicity, and mortality.

Relapses were graded as major and minor. Major relapse was defined as any life- or organ-threatening event due to active vasculitis; minor relapse was defined in absence of organ- or life-threatening manifestations, as the recurrence or first appearance of disease activity sufficient to warrant an increase of the prednisone dose to >25 mg/day for patients on a maintenance dose <15 mg/day or more than 100% for maintenance doses ≥15 mg/day [20]. The treatment of relapses was left at the discretion of the treating physician.

Once the 24-month follow-up was completed, we kept following the patients at our clinic. After the study period, the patients were maintained on a prednisone dose of 5 mg/day. We collected information regarding the long-term follow-up of this cohort. During this period the patients were assessed every 6 months.

### Sample size calculation

In this non-inferiority trial, we assumed the following: 35% probability of having a relapse within 24 months of remission; relapse rate difference between-groups of 15% considered to be statistically significant using a two-tailed Fisher’s exact test; drop-out rate of up to 5%. Based on these assumptions, we estimated that 136 patients per group would achieve 80%-power with a level of statistical significance of 0.05.

### Statistical analysis

Statistical analyses were performed using SPSS version 22.0 and GraphPad Prism 5. Continuous data were reported as median (interquartile range, IQR) and compared by the Student’s t test, Mann-Whitney test, Wilcoxon Signed Rank test and Friedman test where appropriate. Relapse rates were compared across different groups using contingency tables and Fisher’s exact test. Time to remission and time to relapse were assessed by Kaplan–Meier survival analysis and the log-rank test was used to compare different groups. The data were analysed following the intention-to-treat principle. A two-sided p value <0.05 was considered statistically significant.

## Results

### Enrolment and patient characteristics

**Fig 1:**
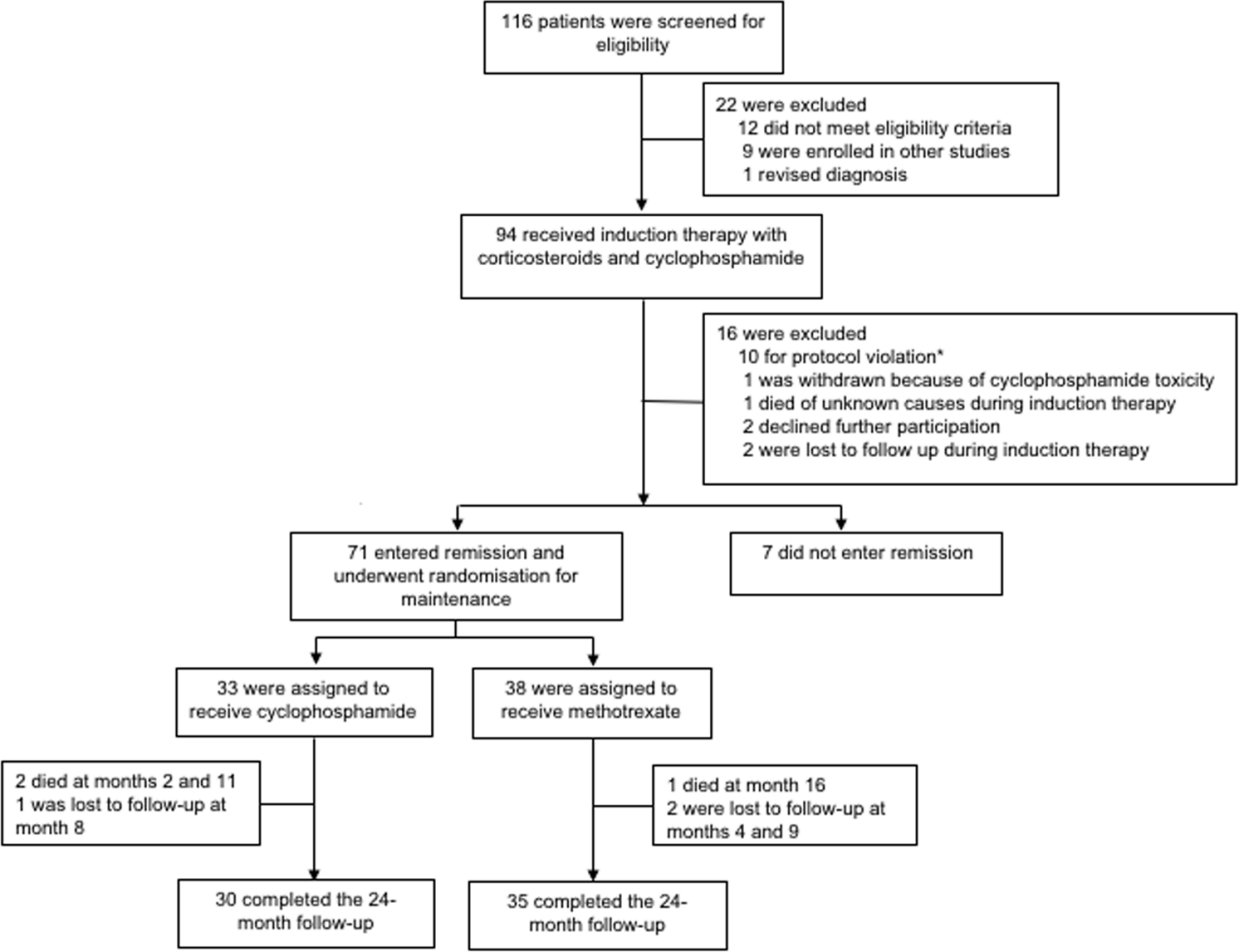
Trial profile. *The patients excluded for protocol violation were judged non-adherent to the induction regimen prescribed.

Between December 1997 and February 2011, 116 patients were screened. Twenty-two patients were not eligible, 94 were enrolled. Sixteen patients (17%) were excluded during the remission-induction phase (Fig 1). Of the remaining 78 patients, seven did not achieve remission within 9 months. Thus, 71 patients were randomised to receive CYC (n = 33) or MTX (n = 38) (Fig 1). The last patient was enrolled in June 2011; the study was closed for analysis in June 2013. The trial was prematurely closed because of the slow enrolment rate and the parallel availability of new treatments for remission maintenance.

Of the 71 randomised patients, 27 (38%) had GPA, 14 (20%) MPA and 30 (42%) EGPA. The diagnosis was biopsy-proven in 40 patients (56%). No statistically significant differences were observed between the CYC and MTX groups with respect to demographic, clinical, laboratory characteristics and BVAS at study entry (Table 1).

**Table 1:** Demographic and clinical characteristics of the patients at study entry.

	CYC (N = 33)	MTX (N = 38)	ALL (N = 71)
Age–median (IQR), years	56 (36–71)	52 (18–77)	54 (18–77)
Sex–no. (%)			
Male	17 (51)	19 (50)	36 (51)
Female	16 (49)	19 (50)	35 (49)
Diagnosis–no. (%)			
GPA	13 (39)	14 (37)	27 (38)
MPA	7 (22)	7 (18)	14 (20)
EGPA	13 (39)	17 (45)	30 (42)
BVAS–median (IQR)	19 (14–24)	18 (10.5–21)	18 (11.5–22.5)
FFS (EGPA)–no. (%)			
FFS = 0	5 (38)	10 (59)	15 (50)
FFS = 1	6 (46)	5 (29)	11 (37)
FFS = 2	2 (16)	2 (12)	4 (13)
Clinical manifestations–no. (%)			
Constitutional symptoms^§^	29 (88)	29 (76)	58 (82)
Ear, nose and throat involvement	31 (94)	34 (89)	65 (92)
Lung involvement	22 (67)	20 (53)	42 (59)
Kidney involvement	15 (45)	10 (26)	25 (35)
Nervous system involvement			
Peripheral	18 (55)	22 (58)	40 (56)
Central	2 (6)	2 (5)	4 (6)
Ocular involvement	6 (18)	7 (18)	13 (18)
Skin involvement	13 (39)	9 (24)	22 (31)
Cardiovascular involvement	7 (21)	4 (11)	11 (15)
Gastrointestinal involvement	4 (12)	4 (11)	8 (11)
ANCA-positive–no. (%)			
By immunofluorescence			
C-ANCA	10 (30)	15 (39)	25 (35)
P-ANCA	19 (58)	15 (39)	34 (48)
By ELISA			
PR3-ANCA	8 (24)	9 (24)	17 (24)
MPO-ANCA	13(39)	13 (34)	26 (37)
Laboratory results–median (IQR)			
Serum creatinine–mg/dl	1.1 (0.8–2.9)	0.9 (0.8–1.2)	1 (0.8–1.5)
eGFR–ml/min	77 (17–91)	84 (62.8–100.3)	79.5 (38–108.9)
C-reactive protein level- mg/L	15.5 (4.7–37)	7.3 (3.9–30.4)	11.5 (4.2–34.4)
ESR–mm/h	52 (15–92)	46.5 (22.8–61.3)	47 (18–74.5)

No statistically significant differences between the CYC and MTX groups were found with respect to all the parameters/characteristics reported in the table

^§^Constitutional symptoms include fatigue, weight loss, anorexia, low-grade fever

CYC, cyclophosphamide; MTX, methotrexate; IQR, interquartile range; GPA, granulomatosis with polyangiitis; MPA, microscopic polyangitiis; EGPA, eosinophilic granulomatosis with polyangiitis; BVAS, Birmingham Vasculitis Score; FFS, Five Factor Score; ANCA, antineutrophil cytoplasmic antibody; C-ANCA, cytoplasmic ANCA; P-ANCA, perinuclear ANCA; PR3-ANCA, proteinase 3-ANCA; MPO-ANCA, myeloperoxidase-ANCA; eGFR, estimated glomerular filtration rate (calculated using the Cockroft-Gault formula); ESR: erythrocyte sedimentation rate

The cumulative CYC dose during the induction phase was 7.0 g (IQR 4.5–11.0) and 8.2 g (IQR 6.0–18.0) in the patients who were later randomised to CYC and MTX, respectively. Eight patients among those randomised to CYC and 5 among those randomised to MTX received CYC dose adjustments due to renal function impairment. The median cumulative CYC dose in patients randomised to CYC during remission maintenance was 25.3 g (IQR 18.5–29.0).

**Fig 2:**
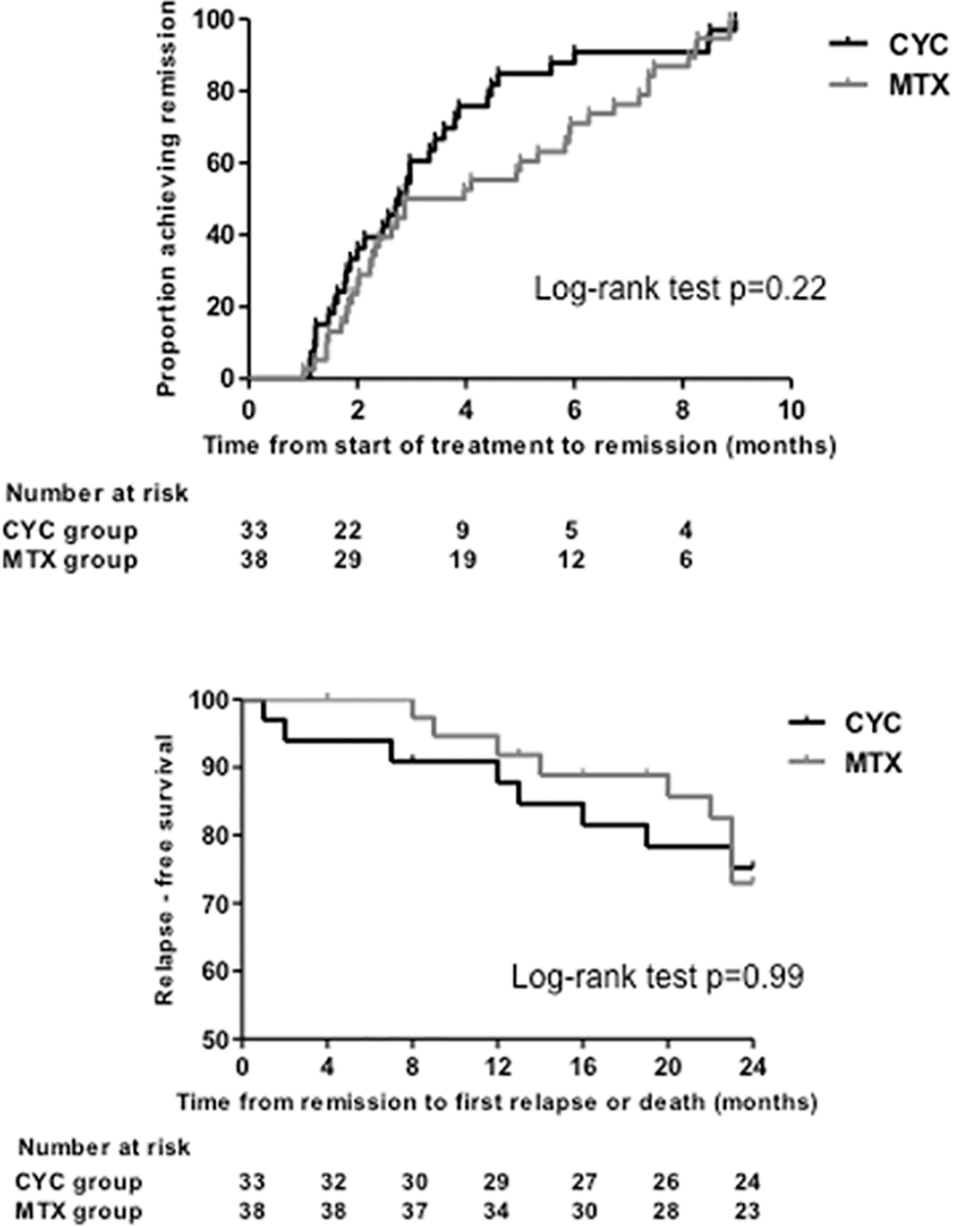
*Upper panel*. Kaplan-Meier estimate of the time from the start of treatment to remission of the patients who were later assigned to receive cyclophosphamide (CYC) or methotrexate (MTX). *Lower panel*. Kaplan-Meier estimate of the time from remission to first relapse or death (during the planned 24 month-follow-up) in the cyclophosphamide (CYC) and methotrexate (MTX) groups.

The time from the start of induction therapy to remission of the patients who were later randomised to receive CYC or MTX was not different (log-rank test p = 0.22) (Fig 2).

### Study end-points

Seven patients in the CYC group and nine in the MTX group experienced relapses during the planned 24-month follow-up after randomisation (p = 1.00). Relapse frequencies at months 12, 18 and 24 were respectively 9%, 15%, 21% and 8%, 11%, 24% in the CYC and MTX groups (p = 1.00, p = 0.73, p = 1.00) (Table 2).

**Table 2:** Relapse frequencies during the planned 24-month follow-up period.

	CYC (N = 33)	MTX (N = 38)	P value*
Relapse (all patients)			
12 mo	3 (9)	3 (8)	1.00
18 mo	5 (15)	4 (11)	0.73
24 mo	7 (21)	9 (24)	1.00
Major relapse	5 (71)	5 (56)	0.75
Minor relapse	2 (29)	4 (44)	0.65
Relapse (GPA + MPA)	**(N = 20)**	**(N = 21)**	1.00
12 mo	2 (10)	2 (10)	1.00
18 mo	3 (15)	3 (14)	0.73
24 mo	4 (20)	6 (29)	
Relapse (EGPA)	**(N = 13)**	**(N = 17)**	
12 mo	1 (8)	1 (6)	1.00
18 mo	2 (15)	1 (6)	0.68
24 mo	3 (23)	3 (18)	1.00

CYC, cyclophosphamide; MTX, methotrexate; GPA, granulomatosis with polyangiitis; MPA, microscopic polyangitiis; EGPA, eosinophilic granulomatosis with polyangiitis

*P values were not corrected for multiple comparisons.

The median relapse-free survival was 24 months (23–24) in the CYC group and 24 months (IQR 19.25–24) in the MTX group (log-rank test p = 0.99) (Fig 2). There were five major and two minor relapses in the CYC group and five major and four minor in the MTX group (p = 0.75 and p = 0.65). The median BVAS at the time of the relapse was 11 (IQR 7.5–18.5) in the CYC and 11 (IQR 8–12) in the MTX group (p = 0.28).

There were no significant differences between the CYC and MTX groups with respect to renal function and proteinuria at the end of maintenance therapy (month 12) and at the end of month 24 (S1 Table). Within each treatment group, no statistically significant change in eGFR during the study period were found. Conversely, in both groups there was a progressive reduction in proteinuria, which was statistically significant only in the CYC group (S1 Appendix); also the slope in proteinuria between study entry and month 24 was significantly greater in the CYC group (S2 Appendix). At the end of the follow-up, only a few patients (three in the CYC arm and two in the MTX arm) developed chronic kidney disease (CKD) grade 4–5, while none developed end-stage renal disease (S1 Table).

### Deaths/Adverse events

Three patients died during the 24-month study period. Two were in the CYC group: one died for a severe disease flare (month 2 after randomisation), and one for aspiration pneumonia (month 11). One patient (MTX group) died for B-cell lymphoma (month 16). Three patients were lost to follow-up: one in the CYC group (month 8) and two in the MTX group (respectively at months 4 and 9).

In addition to the adverse events leading to death, other adverse events were recorded; the events that occurred during the planned 24-month follow-up are reported in Table 3. Leukopenia was more frequent in the CYC than in the MTX group, although the difference was not statistically significant (p = 0.16); all these cases were successfully managed with drug reduction. On the other hand, infections (of mild-to-moderate severity) were slightly more frequent in the MTX group (p = 0.16). Overall, there were no significant differences in the percentages of adverse events of any type in the two groups (Table 3).

**Table 3:** Adverse events during the planned 24-month follow-up period.

Adverse events	Mild/moderate^¶^			Severe/life-threatening^¶^		
	CYC N = 33	MTX N = 38	*P value*	CYC N = 33	MTX N = 38	*P value*
Leukopenia	7 (21)	3(8)	0.16	0 (0)	0 (0)	1.00
Diabetes	2 (6)	3 (8)	0.78	0 (0)	0 (0)	1.00
Infection	2 (6)	7 (18)	0.16	1 (3)	2 (5)	0.89
Osteoporosis	8 (24)	8 (21)	0.79	1 (3)	1 (3)	1.00
Cataract	5 (15)	3 (8)	0.39	0 (0)	0 (0)	1.00
Gastrointestinal	1(3)	1 (3)	1.00	0 (0)	0 (0)	1.00
Cardiovascular	3 (9)	1 (3)	0.26	1 (3)	0 (0)	0.19
Other	2 (6)	6 (16)	0.24	1 (3)	2 (5)	0.65

^¶^ Mild/moderate and severe/life-threatening respectively correspond to grades 1–2 and 3–4 following the National Cancer Institute Common Terminology Criteria for Adverse Events, version 3. Adverse events leading to death were not included in this table (see text for details)

CYC, cyclophosphamide; MTX, methotrexate

We observed no MTX-related renal toxicity, both in the entire MTX group and in the five patients with an eGFR of 30–50 mL/min at the time of randomisation.

### Extended follow-up

Of the 55 patients who were in remission at month 24, 42 (24 in the CYC and 18 in the MTX arm) continued their follow-up at our centre. Their median follow-up after month 24 was 37.8 months (IQR 11.6–65.4). In this extended follow-up period, six patients in the CYC group and three in the MTX group relapsed.

The median overall follow-up (study period+extended follow-up) was 55.6 months (IQR 26.5–87.8); during the overall follow-up, thirteen patients in the CYC group and twelve in the MTX group had relapses (39.4% and 31.6% respectively); overall, 19 relapses were recorded in the CYC and 16 in the MTX group. The number of patients with multiple relapses (two or more) during the overall follow up was four in the CYC group and three in the MTX group. The median time to first relapse or death was 23 months (IQR 13–44) in the CYC group and 22.5 months (IQR 13.5–23.75) in the MTX group (log-rank test p = 0.94) (S3 Appendix*)*.

During the extended follow-up, one patient (MTX group) developed bladder cancer, and two patients died, one at month 96 for unknown cause (CYC group) and one at month 112 for congestive heart failure (MTX group).

### Subgroup analysis

**Fig 3:**
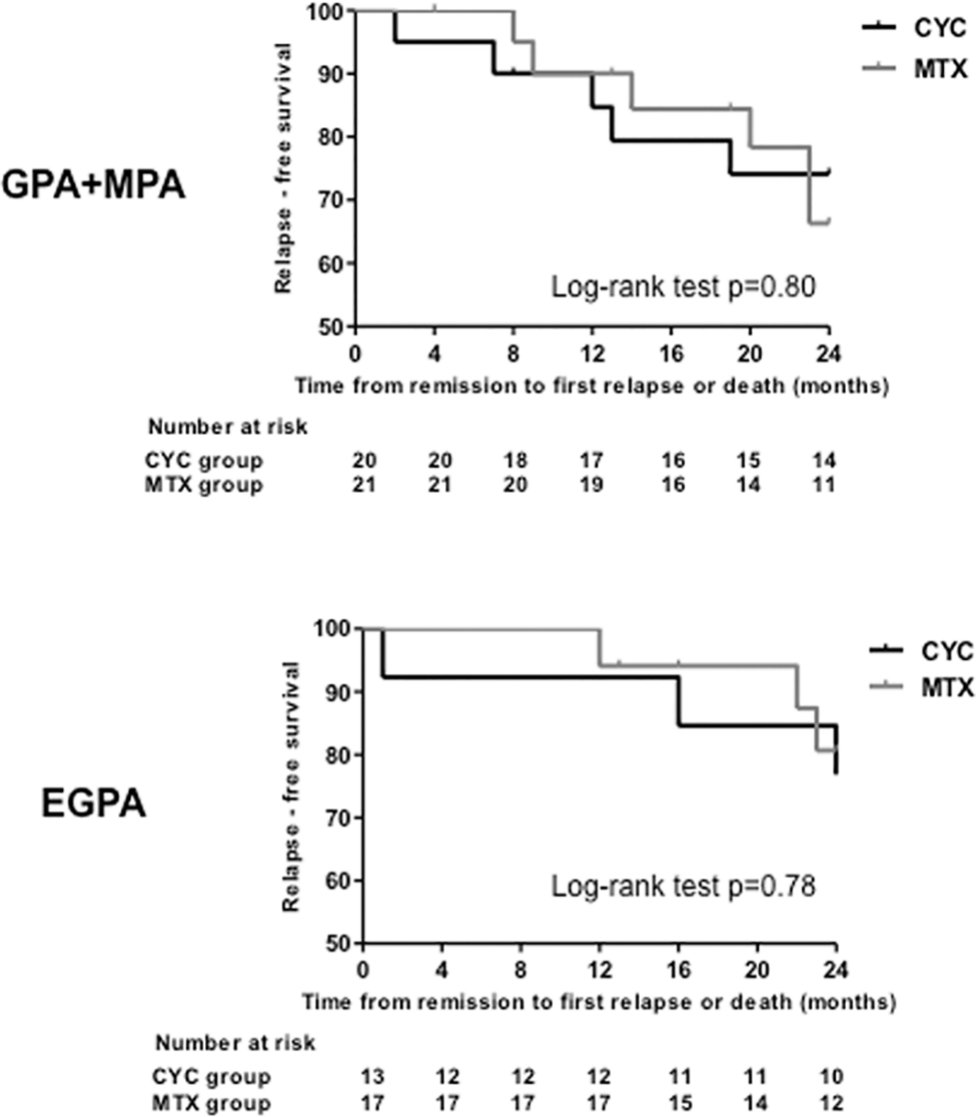
Subgroup analysis of the Kaplan-Meier estimate of the time from remission to first relapse or death (during the planned 24 month-follow-up) in the cyclophosphamide (CYC) and methotrexate (MTX) groups. The *upper panel* shows the analysis in patients with GPA or MPA (granulomatosis with polyangiitis or microscopic polyangiitis) while the *lower panel* shows the analysis in patients with EGPA (eosinophilic granulomatosis with polyangiitis).

We analysed relapses in the GPA+MPA and EGPA subgroups. When considering GPA+MPA, four relapses occurred in the CYC group and six in the MTX group during the study period (p = 0.73) (Table 2). There was no statistically significant difference in relapse-free survival between the two groups (log-rank test p = 0.80) (Fig 3).

When considering EGPA, three relapses were recorded in the CYC group and three in the MTX group (p = 1.00) (Table 2). The relapse-free survival was similar in the two groups (log-rank test p = 0.78) (Fig 3).

The time to relapse over the whole follow-up (study period+extended follow-up) was comparable between the CYC and MTX arms when the GPA+MPA and EGPA subgroups were analysed separately (S4 Appendix and S5 Appendix).

## Discussion

In this randomised trial of MTX versus CYC for remission maintenance in AAV, the efficacy in preventing relapses of the two study drugs was comparable, and both treatments led to good renal outcomes and overall survival in the long-term follow-up. This study differed from previous trials [10–12] for the inclusion of EGPA patients, who were enrolled if they had poor-prognosis factors or neuropathy. The analysis of relapse rates, relapse-free survival and overall survival in the GPA+MPA versus EGPA subgroups also showed similar efficacy of CYC and MTX.

The recent advances in the management of AAV have dramatically changed its prognostic profile. While AAV was initially characterised by significant short-term mortality [21], it is now considered as a chronic-relapsing disorder whose long-term prognosis is mainly influenced by chronic organ damage, cardiovascular disease and treatment-related toxicity [22, 23]. This emphasizes the need for effective and safe remission-maintenance agents, able to reduce the risk of relapses while carrying an acceptable toxicity profile. In addition, as such therapies are usually given for prolonged periods, drug-related costs must also be considered.

Different controlled trials have focussed on remission maintenance in AAV; among these, only the WEGENT trial tested MTX (against azathioprine). This trial included GPA and MPA patients and showed that MTX and azathioprine had comparable efficacy and similar toxicity. The primary end-point was an event causing the discontinuation of the study drug or death. Remission was induced using CYC pulses. The primary end-point was reached in 11% and 19% of the azathioprine- and MTX-treated patients respectively, this difference being non-statistically significant. Relapses occurred in 37% and 33% of the patients who received azathioprine or MTX [11]. The relapse rates recorded in our study are lower than those in the WEGENT trial; although several factors may account for this difference, the exposure to CYC during induction was probably higher in our study than in the WEGENT. Unlike in the WEGENT trial, none of our patients had to withdraw MTX due to toxicity, which may be due to the lower target dose of MTX we used (20 mg/week instead of 25 mg/week) [11].

Maintenance studies in AAV report relapse rates that are difficult to compare with ours, mainly due to differences in treatment duration and follow-up. The CYCAZAREM trial, comparing CYC and azathioprine, showed 18-month relapse rates of 15.5% and 13.7% in the CYC and azathioprine groups, respectively.[10] These rates were very similar to those obtained in our study at 18 months. The IMPROVE trial, comparing azathioprine and mycophenolate mofetil, reported 42-month relapse rates of 37.% and 55.2% in the azathioprine and mycophenolate groups.[12] Finally, the MAINRITSAN trial, comparing azathioprine and rituximab, showed 28-month relapse rates of 29% and 5% in the azathioprine and rituximab groups, respectively. [13] Survival rates were quite similar across the above studies and, as in our trial, were generally lower than 5%.

Overall, our trial showed that MTX is safe, and that it can be a good candidate for long-term maintenance therapy as it was demonstrated to be in other autoimmune disorders, particularly rheumatoid arthritis [24]. We also showed that MTX is safe in patients with mild-to-moderate renal disease. In our study, patients could be assigned to receive MTX (or CYC) if they had an eGFR>30 mL/min at randomisation. MTX was as effective as CYC in preserving renal function during the whole follow-up, and no patient had to withdraw the drug or reduce its dosage because of suspected renal toxicity. However, the decline in proteinuria was significant only in the CYC group.

A major point of our trial relates to the inclusion of EGPA patients. EGPA has never been grouped together with GPA and MPA in randomised trials, essentially because of a different initial response to immunosuppressive therapies. EGPA, in fact, can be successfully managed even with glucocorticoids alone, although it is widely accepted that immunosuppressive drugs are needed in the presence of renal, gastrointestinal, cardiac or central nervous system involvement (*i*.*e*., poor-prognosis factors). Peripheral neuropathy, which is often debilitating in EGPA [25], also requires immunosuppression[17]. Only two randomised trials have been published so far in EGPA: in the first, which included patients with poor-prognosis factors, a 12 CYC pulse-regimen was superior than a 6-pulse one in inducing sustained remission [5]. In the second, enrolling patients without poor-prognosis factors initially treated with glucocorticoid monotherapy, one third of the cases eventually needed immunosuppressive agents, with pulse CYC showing similar efficacy as compared with azathioprine.[6] None of these two trials, however, differentiated an induction and a maintenance phase. Our study, albeit on a limited sample size (overall, 30 EGPA patients were randomised) is the first to demonstrate the feasibility of a staged induction-maintenance approach in EGPA; this finding is clinically relevant, because prevention of relapses and reduction of glucocorticoid exposure are major issues in the management of EGPA [26, 27].

Although AAVs may have distinct clinical courses and response to therapy, we could not detect significant differences between CYC and MTX in maintaining remission when GPA and MPA were compared with EGPA. This finding, however, warrants replication in larger studies.

Our trial certainly has important limitations. First, the sample size is small, which does not allow us to draw firm conclusions regarding the comparable efficacy of MTX and CYC. Second, patients were not stratified on disease severity, as recent trials did. Third, the enrolment period was particularly long, thus changes in screening and diagnostic tools over time might have influenced patient management. Importantly, alternative maintenance agents such as azathioprine became available after the publication of the CYCAZAREM trial [10]. However, the results of this trial did not demonstrate superior safety of azathioprine over CYC, and the long-term follow-up data [28] had not yet been published when we closed the study.

In conclusion, MTX appears an effective and safe maintenance agent in AAV, also in the long term. Similar to GPA and MPA, EGPA patients with poor-prognosis factors may benefit from a staged induction-maintenance approach.

## Supporting information

S1 Table Renal outcomes during the study period. (DOCX)

S1 Appendix Changes in estimated glomerular filtration rates (eGFR) and proteinuria during the study period in the cyclophosphamide (CYC) and methotrexate (MTX) groups. (TIFF)

S2 Appendix Proteinuria slope (expressed as reciprocal of the ratio between the baseline value and the value at given time-points of proteinuria) in the two groups. The slope was significantly greater in the CYC than in the MTX group (**p<0.01) in the time-interval comprised between diagnosis and month 24.
(TIFF)

S3 Appendix Kaplan-Meier estimate of the time from remission to first relapse or death in the cyclophosphamide (CYC) and methotrexate (MTX) groups during the whole follow-up (planned 24-month study period+ extended follow-up). The dotted line indicates the planned 24-month follow-up.
(TIFF)

S4 Appendix Kaplan-Meier estimate of the time from remission to first relapse or death during the whole follow-up of the study in the subgroup of patients with GPA or MPA (granulomatosis with polyangiitis or microscopic polyangiitis). The dotted line indicates the planned 24-month follow-up.
(TIFF)

S5 Appendix Kaplan-Meier estimate of the time from remission to first relapse or death during the whole follow-up of the study in the subgroup of patients with EGPA (eosinophilic granulomatosis with polyangiitis). The dotted line indicates the planned 24-month follow-up.
(TIFF)

S1 Checklist Consort checklist. Consort checklist of Powecime trial.
(DOC)

S1 Protocol Powercime protocol. Protocol of Powercime trial.
(PDF)
