# Supplementary material for: Methotrexate versus cyclophosphamide for remission maintenance in ANCA-associated vasculitis: A randomised trial
Source: PLoS One. 2017 Oct 10;12(10):e0185880. doi: 10.1371/journal.pone.0185880 (PMC5634660; doi:10.1371/journal.pone.0185880)
Supplement: S1 Table — (DOCX) [file pone.0185880.s001.docx]

**S1 Table.** Renal outcomes during the study period

|  | **CYC**  **(N = 33)** | **MTX**  **(N = 38)** | ***P value*** | |
| --- | --- | --- | --- | --- |
| sCreat (mg/dL), median (IQR)  diagnosis  randomisation  12 mo  24 mo | 1.1 (0.8 – 2.9)  1.0 (0.8 – 1.4)  1.0 (0.8 – 1.2)  0.95 (0.8 – 1.3) | 0.9 (0.8 – 1.2)  0.9 (0.8 – 1.1)  0.9 (0.8 – 1.1)  0.9 (0.7 – 1.1) | | 0.25  0.37  0.20  0.35 |
| eGFR (mL/min), median (IQR)  diagnosis  randomisation  12 mo  24 mo | 77 (17 – 91)  80 (44 – 101)  83 (65.5 – 90.5)  82.5 (59.5 – 95.5) | 84 (62.75 – 100.3)  89 (75.25 – 99.3)  87 (70 – 106.3)  86 (75.5 – 98.8) | | 0.12  0.36  0.18  0.44 |
| proteinuria (mg/24h), median (IQR)  diagnosis  randomisation  12 mo  24 mo | 275 (146.75 – 1275)  145 (68.25 – 415.5)  78 (51 – 204)  66.5 (30 – 112) | 150 (72 – 445)  110 (55 – 325)  90 (30 – 172.5)  139 (50 – 240.5) | | 0.16  0.32  0.42  0.09 |
| CKD stage 4-5, no.(%)  12 mo  24 mo  end of f-up | 1 (3)  3 (9)  3 (9) | 2 (5)  2 (5)  2 (5) | | 0.64  0.53  0.53 |

CYC, cyclophosphamide; MTX, methotrexate; sCreat, serum creatinine; IQR, interquartile range; eGFR, estimated glomerular filtration rate; CKD, chronic kidney disease
