# Supplementary figures and images for: Methotrexate versus cyclophosphamide for remission maintenance in ANCA-associated vasculitis: A randomised trial
Source: PLoS One. 2017 Oct 10;12(10):e0185880. doi: 10.1371/journal.pone.0185880 (PMC5634660; doi:10.1371/journal.pone.0185880)

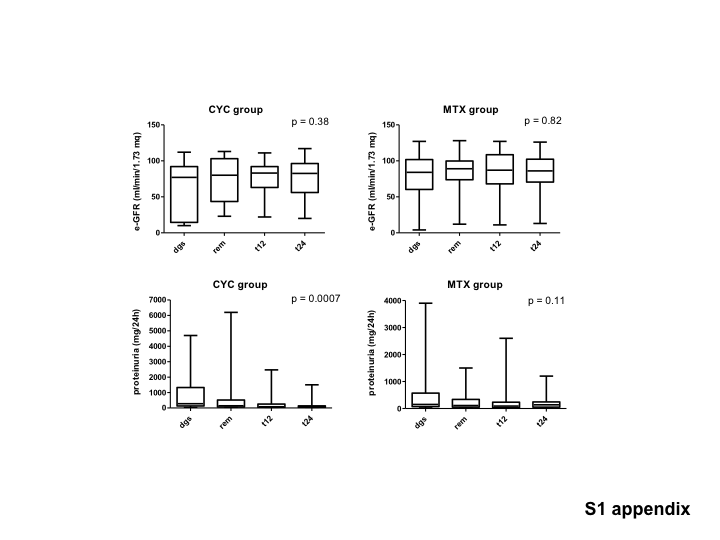

Supplement: S1 Appendix — (TIFF) [file pone.0185880.s002.tiff]

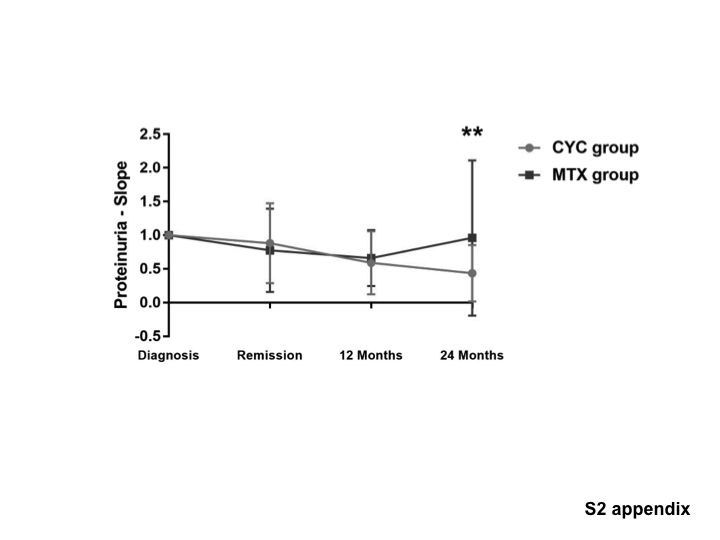

Supplement: S2 Appendix — The slope was significantly greater in the CYC than in the MTX group (**p<0.01) in the time-interval comprised between diagnosis and month 24. (TIFF) [file pone.0185880.s003.tiff]

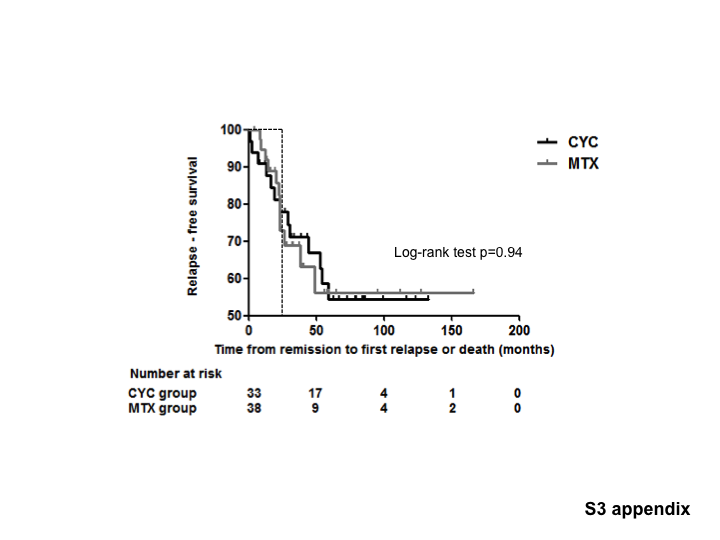

Supplement: S3 Appendix — The dotted line indicates the planned 24-month follow-up. (TIFF) [file pone.0185880.s004.tiff]

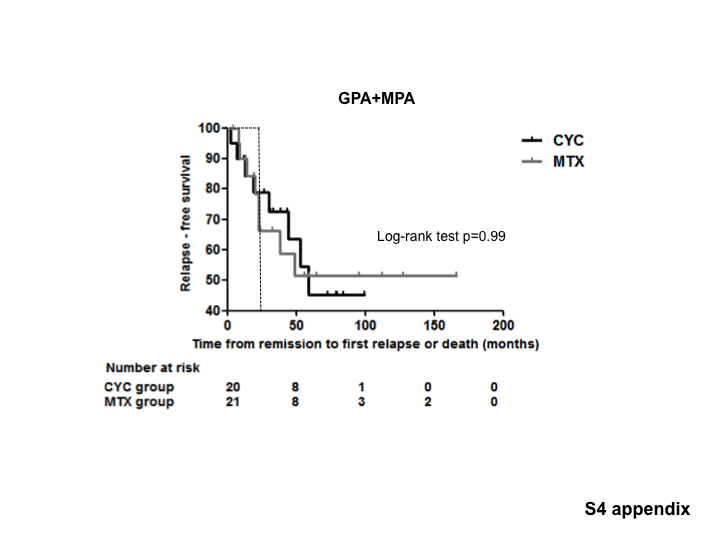

Supplement: S4 Appendix — The dotted line indicates the planned 24-month follow-up. (TIFF) [file pone.0185880.s005.tiff]

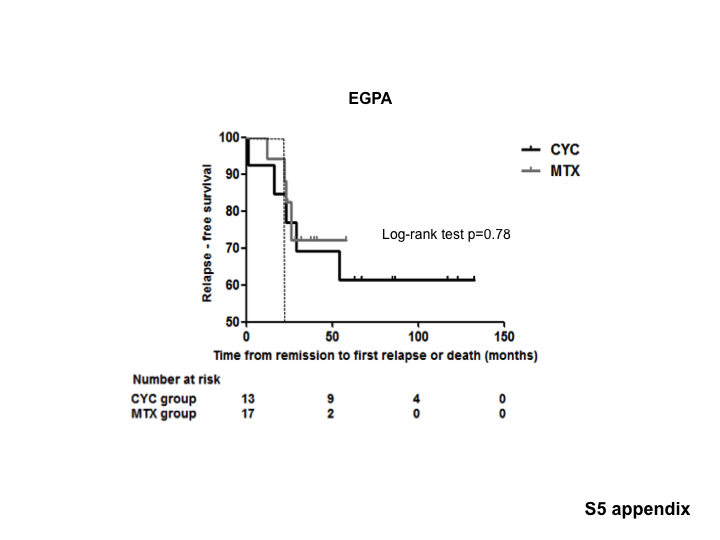

Supplement: S5 Appendix — The dotted line indicates the planned 24-month follow-up. (TIFF) [file pone.0185880.s006.tiff]
