# Supplementary material for: Methotrexate versus cyclophosphamide for remission maintenance in ANCA-associated vasculitis: A randomised trial
Source: PLoS One. 2017 Oct 10;12(10):e0185880. doi: 10.1371/journal.pone.0185880 (PMC5634660; doi:10.1371/journal.pone.0185880)
Supplement: S1 Protocol — Protocol of Powercime trial. (PDF) [file pone.0185880.s008.pdf]

Protocol: METHOTREXATE VERSUS CYCLOPHOSPHAMIDE FOR REMISSION MAINTENANCE IN ANCA-ASSOCIATED VASCULITIS

POWERCIME TRIAL

Version 1.2- June 1997

Principal Investigator

Dr Carlo Buzio
Unità Operativa di Nefrologia,
Azienda Ospedaliero-Universitaria di Parma
Via Gramsci 14,
43100 Parma (ITALY)
tel: +39 0521 702345; 702013

Local collaborators

Dr Alberto Pesci
Clinica Tisiopneumologica
Azienda Ospedaliero-Universitaria di Parma
Via Gramsci 14,
43100 Parma (ITALY)

Dr Laura Pavone
Unità Operativa di Nefrologia,
Azienda Ospedaliero-Universitaria di Parma
Via Gramsci 14,
43100 Parma (ITALY)

Other participating centres/investigators

Dr Simeone Andrulli
Divisione di Nefrologia
Azienda Ospedaliera della Provincia di Lecco
Via dell'Eremo, 9/11
23900 Lecco (ITALY)

Dr Bruno Tumiati
Unità Operativa di Medicina Interna
Arcispedale Santa Maria Nuova
Viale Umberto I, 50
42100 Reggio Emilia (ITALY)

1. BACKGROUND AND AIM OF THE STUDY

ANCA associated vasculitis (AAVs) includes Wegener's Granulomatosis (WG), microscopic polyangitiis (MPA) and Churg–Strauss Syndrome (CSS) [1, 2]. These disorders are characterized by necrotizing

vasculitis of the small vessels with predilection for the kidneys, lungs and peripheral nervous system. Rapidly progressive glomerulonephritis and diffuse alveolar hemorrhage are the most severe clinical manifestations of WG and MPA [3]. These syndromes are associated with the positivity of ANCA autoantibodies in 90% of patients with WG and MPA and in 40% of CSS [4, 5].

Standard therapy to induce remission in ANCA-associated vasculitis is based on the combination of corticosteroids and cyclophosphamide (CYC) in order to treat or prevent vital organ dysfunction induced by active vasculitis [6]. This induction regimen is effective in 70 – 90% of MPA and WG. However, the limits of this approach are the high frequency of recurrent disease, especially during the reduction or discontinuation of therapy, and an increased incidence of malignancy and infections.

In a prospective study including 158 WG patients treated with CYC and corticosteroids, 50% of patients that achieved remission relapsed between 3 months and 16 years after remission [7]. In a retrospective study including 150 vasculitis patients, the relapse rate was 34% [8].

Moreover, Hoffman at al found that 42% of patients had treatment-related serious adverse events. Adverse events related to CYC were infections (46%), haemorrhagic cystitis (43%), bladder cancer (2%), and myelodysplasia (2%). Side effects of corticosteroids were cataract (21%), bone fractures (11%) and aseptic bone necrosis (3%) [7]. The risk of bladder cancer induced by CYC has been confirmed by many authors [9].

MTX has been successfully used as induction therapy in rheumatoid arthritis, Takayasu arteritis, panarteritis nodosa [10-12]. Uncontrolled and pilot studies reported the efficacy and safety of MTX for the treatment of patients with AAV both for induction and maintenance [13, 14].

In this open-label randomised trial we will compare the efficacy and safety of MTX and CYC as maintenance treatment in patients with WG, MPA and poor–prognosis CSS after remission induction with oral CYC.

2. ELIGIBILITY CRITERIA

Inclusion criteria

- Written signed informed consent
- Clinically active AAV (either newly diagnosed or relapsing/refractory)
- Age 18-80 years
- Life-expectancy > 1 year

Exclusion criteria

- creatinine clearance < 10 ml/min/1.73 mq
- aminotransferase levels more than twice the upper limit of the normal range
- chronic viral infections (HIV, HBV, HCV)
- coexistence of connective tissue disease
- documented contraindication to prednisolone, cyclophosphamide or methotrexate
- pregnancy
- concurrent malignancies or malignant neoplasms that occurred during the 5 years prior to enrolment (with the exception of adequately treated non-melanoma skin cancers)

3. DIAGNOSTIC CRITERIA

Patients with WG or CSS have to fulfill the 1990 American College of Rheumatology criteria and/or the 1994 Chapel Hill Consensus Conference definitions. Patients with MPA have to meet the 1994 Chapel Hill definitions[1, 2]. CSS prognosis will be assessed according to the five-factor score (FFS); only patients with FFS≥1 or with peripheral neuropathy will be included [15].

4. STUDY DRUG ADMINISTRATION, RANDOMISATION MODALITY AND CONCOMITANT TREATMENTS

All the patients included in the study will receive the same induction therapy, consisting of three IV infusions of methylprednisolone (500 or 1000 mg, depending on the body weight) followed by oral prednisone and oral CYC. Prednisone will be given at the dose of 1 mg/kg/day for the first month, 0.5 mg/kg/day for month 2, 0.25 mg/kg/day for month 3, and then tapered to 5 mg/day by month 6. CYC will be administered at daily oral dosage of 2 mg/kg/day. Remission is defined as a Birmingham Vasculitis Activity Score (BVAS) of 0 (i.e., the absence of signs of disease activity) [16]. Patients who achieve remission will be randomly assigned to receive maintenance therapy with CYC or MTX. Randomisation will be performed by Dr Andrulli using a computer algorithm concealed from the other investigators. The patients will be randomised to CYC or MTX at a 1:1 ratio. For maintenance, CYC will be given at the dose of 1.5 mg/kg/day while MTX at the dose of 15 mg/week initially, progressively increased at a weekly rate of 2.5 mg until the maximum dose of 0.3 mg/kg/week is achieved. Patients with GFR between 10 and 50 ml/min/1.73 mq will receive MTX at half dose. CYC group will receive prophylaxis with trimethoprim-sulfamethoxazole at dosage of 80/400 mg/day to prevent *Pneumocystis carinii* pneumonia; MTX group will receive weekly oral folic acid (5 mg). Maintenance treatment will be continued for 12-months after which MTX or CYC will be discontinued and the patients will be followed up for at least further 24 months. Prednisone will be instead continued at 5 mg/day.

5. ASSESSMENT OF EFFICACY AND TREATMENT-RELATED TOXICITY

Disease response will be assessed every month before the randomization and then every three months. During the post treatment follow up the patients will be assessed every 3 – 6 months. Disease response evaluation includes a BVAS assessment and routine laboratory test, such as full blood counts, renal and liver function, C-reactive protein (CRP), erythrocyte sedimentation rate (ESR), ANCA test, urinalysis and 24 – hour proteinuria.

6. STUDY END-POINTS

Primary end-point

- relapse rate by month 12 (after remission)
- time from remission to relapse

Secondary end-points

- major and minor relapse rates (with major relapse defined as any life- or organ-threatening event due to active vasculitis and minor relapse defined by the recurrence or first appearance of disease activity sufficient to warrant an increased prednisone dose to >25 mg/day for patients on a maintenance dose <15 mg/day or more than 100% for maintenance doses ≥15 mg/day, without organ or life-threatening manifestations)
- change in eGFR and proteinuria
- therapy-related toxicity
- mortality

7. SAMPLE SIZE CALCULATION

In this non-inferiority trial, we assume the following: 35% probability of having a relapse within 24 months of remission; relapse rate difference between-groups of 15% considered to be statistically significant using a two-tailed Fisher's exact test; drop-out rate of up to 5%. Based on these assumptions, we estimate that 136 patients per group would achieve 80%-power with a level of statistical significance of 0.05.

8. DATA COLLECTION AND STATISTICAL ANALYSIS

During each visit, patients' data will initially be recorded by hand by the investigator(s) on paper and then entered into an electronic data management file/case report form (CRF). The CRF will be created by a specialized agency. Continuous data will be reported as median (interquartile range, IQR) and compared by the Student's t test, Mann-Whitney test, Wilcoxon Signed Rank test and Friedman test where appropriate. Relapse rates will be compared across different groups using contingency tables and Fisher's exact test. Time to remission and time to relapse will be assessed by Kaplan–Meier survival analysis and the log-rank test will be used to compare the two groups. We will analyse data following the intention-to-treat principle. A two-sided p value <0.05 will be considered statistically significant.

9. PUBLICATION OF THE RESULTS

The final manuscript will be submitted to peer-reviewed journals in the field of internal medicine, rheumatology or nephrology. The author list will include the principal investigator, the local collaborators and the other investigators who collaborated to protocol development and to study completion.

REFERENCES

1. Hunder, G.G., et al., *The American College of Rheumatology 1990 criteria for the classification of vasculitis. Introduction.* Arthritis Rheum, 1990. **33**(8): p. 1065-7.
2. Jennette, J.C., et al., *Nomenclature of systemic vasculitides. Proposal of an international consensus conference.* Arthritis Rheum, 1994. **37**(2): p. 187-92.
3. Gross, W.L., *Systemic necrotizing vasculitis.* Baillieres Clin Rheumatol, 1997. **11**(2): p. 259-84.
4. Leavitt, R.Y., et al., *The American College of Rheumatology 1990 criteria for the classification of Wegener's granulomatosis.* Arthritis Rheum, 1990. **33**(8): p. 1101-7.
5. Masi, A.T., et al., *The American College of Rheumatology 1990 criteria for the classification of Churg-Strauss syndrome (allergic granulomatosis and angiitis).* Arthritis Rheum, 1990. **33**(8): p. 1094-100.
6. Luqmani, R.A., et al., *Disease assessment and management of the vasculitides.* Baillieres Clin Rheumatol, 1997. **11**(2): p. 423-46.
7. Hoffman, G.S., et al., *Wegener granulomatosis: an analysis of 158 patients.* Ann Intern Med, 1992. **116**(6): p. 488-98.
8. Gordon, M., et al., *Relapses in patients with a systemic vasculitis.* Q J Med, 1993. **86**(12): p. 779-89.
9. Talar-Williams, C., et al., *Cyclophosphamide-induced cystitis and bladder cancer in patients with Wegener granulomatosis.* Ann Intern Med, 1996. **124**(5): p. 477-84.
10. Nordstrom, D.M., et al., *Pulse methotrexate therapy in rheumatoid arthritis. A controlled prospective roentgenographic study.* Ann Intern Med, 1987. **107**(6): p. 797-801.
11. Hoffman, G.S., et al., *Treatment of glucocorticoid-resistant or relapsing Takayasu arteritis with methotrexate.* Arthritis Rheum, 1994. **37**(4): p. 578-82.
12. Calabrese, L.H., G.S. Hoffman, and L. Guillevin, *Therapy of resistant systemic necrotizing vasculitis. Polyarteritis, Churg-Strauss syndrome, Wegener's granulomatosis, and hypersensitivity vasculitis group disorders.* Rheum Dis Clin North Am, 1995. **21**(1): p. 41-57.
13. de Groot, K., et al., *Therapy for the maintenance of remission in sixty-five patients with generalized Wegener's granulomatosis. Methotrexate versus trimethoprim/sulfamethoxazole.* Arthritis Rheum, 1996. **39**(12): p. 2052-61.
14. Sneller, M.C., et al., *An analysis of forty-two Wegener's granulomatosis patients treated with methotrexate and prednisone.* Arthritis Rheum, 1995. **38**(5): p. 608-13.
15. Guillevin, L., et al., *Prognostic factors in polyarteritis nodosa and Churg-Strauss syndrome. A prospective study in 342 patients.* Medicine (Baltimore), 1996. **75**(1): p. 17-28.

16. Luqmani, R.A., et al., *Birmingham Vasculitis Activity Score (BVAS) in systemic necrotizing vasculitis*. QJM, 1994. **87**(11): p. 671-8.
